# Genomic impact of eukaryotic transposable elements

**DOI:** 10.1186/1759-8753-3-19

**Published:** 2012-11-21

**Authors:** Irina R Arkhipova, Mark A Batzer, Juergen Brosius, Cédric Feschotte, John V Moran, Jürgen Schmitz, Jerzy Jurka

**Affiliations:** 1 Josephine Bay Paul Center for Comparative Molecular Biology and Evolution, Marine Biological Laboratory, Woods Hole, MA, 02543, USA; 2 Department of Biological Sciences, Louisiana State University, 202 Life Sciences Bldg, Baton Rouge, LA, 70803, USA; 3 Institute of Experimental Pathology, ZMBE, University of Münster, Münster D-48149, Germany; 4 Department of Human Genetics, University of Utah, 15 North 2030 East, Rm. 2100, Salt Lake City, UT, 84112, USA; 5 Departments of Human Genetics and Internal Medicine, Howard Hughes Medical Institute, The University of Michigan Medical School, Ann Arbor, MI, 48109-5618, USA; 6 Genetic Information Research Institute, 1925 Landings Drive, Mountain View, CA, 94043, USA

## Abstract

The third international conference on the genomic impact of eukaryotic transposable elements (TEs) was held 24 to 28 February 2012 at the Asilomar Conference Center, Pacific Grove, CA, USA. Sponsored in part by the National Institutes of Health grant 5 P41 LM006252, the goal of the conference was to bring together researchers from around the world who study the impact and mechanisms of TEs using multiple computational and experimental approaches. The meeting drew close to 170 attendees and included invited floor presentations on the biology of TEs and their genomic impact, as well as numerous talks contributed by young scientists. The workshop talks were devoted to computational analysis of TEs with additional time for discussion of unresolved issues. Also, there was ample opportunity for poster presentations and informal evening discussions. The success of the meeting reflects the important role of Repbase in comparative genomic studies, and emphasizes the need for close interactions between experimental and computational biologists in the years to come.

## Introduction

The diversity of topics and focal areas presented at the 2012 Asilomar conference on the “Genomic Impact of Eukaryotic Transposable Elements” was remarkable. Overall the conference had a variety of foci ranging from the biology of transposable elements (TEs) to how the host responds to their impact upon the genome. In addition, a number of valuable new tools were presented that will aid in the detection and characterization of transposons within sequenced genomes. The organizer’s decision to give much stage presence to junior scientists was commendable. These talks impressed the audience and were considered among the best and most exciting contributions at the meeting.

### Homage to a pioneer

It is impossible to begin this conference report without paying homage to the towering figure of Roy John Britten, who passed away at the age of 92 on 21 January 2012. Indeed, the conference started with a personal homage to Roy Britten’s life by the organizer, Jerzy Jurka. Roy’s life and scientific contributions inspired many generations of scientists and his work transcended many fields. Roy was trained as a physicist and began his post-doctoral work as a staff member in the Department of Terrestrial Magnetism at the Carnegie Institution of Washington. There he exploited DNA hybridization to make the groundbreaking discovery of eukaryotic repetitive sequences [1]. This discovery led to another classical paper with Eric Davidson, addressing the role of non-coding DNA in gene regulation [2].

As DNA sequences became more available in the 1980s, Roy began systematic sequence studies of repetitive DNA and served as an inspiration to generations of younger scientists. The relationship between repetitive sequences and TEs, originally discovered by Barbara McClintock, was not yet clear. Notably, Roy contributed to the discovery of human Alu subfamilies, which led to the concept of source/master genes equivalent to active TEs [3].

**Figure 1:**
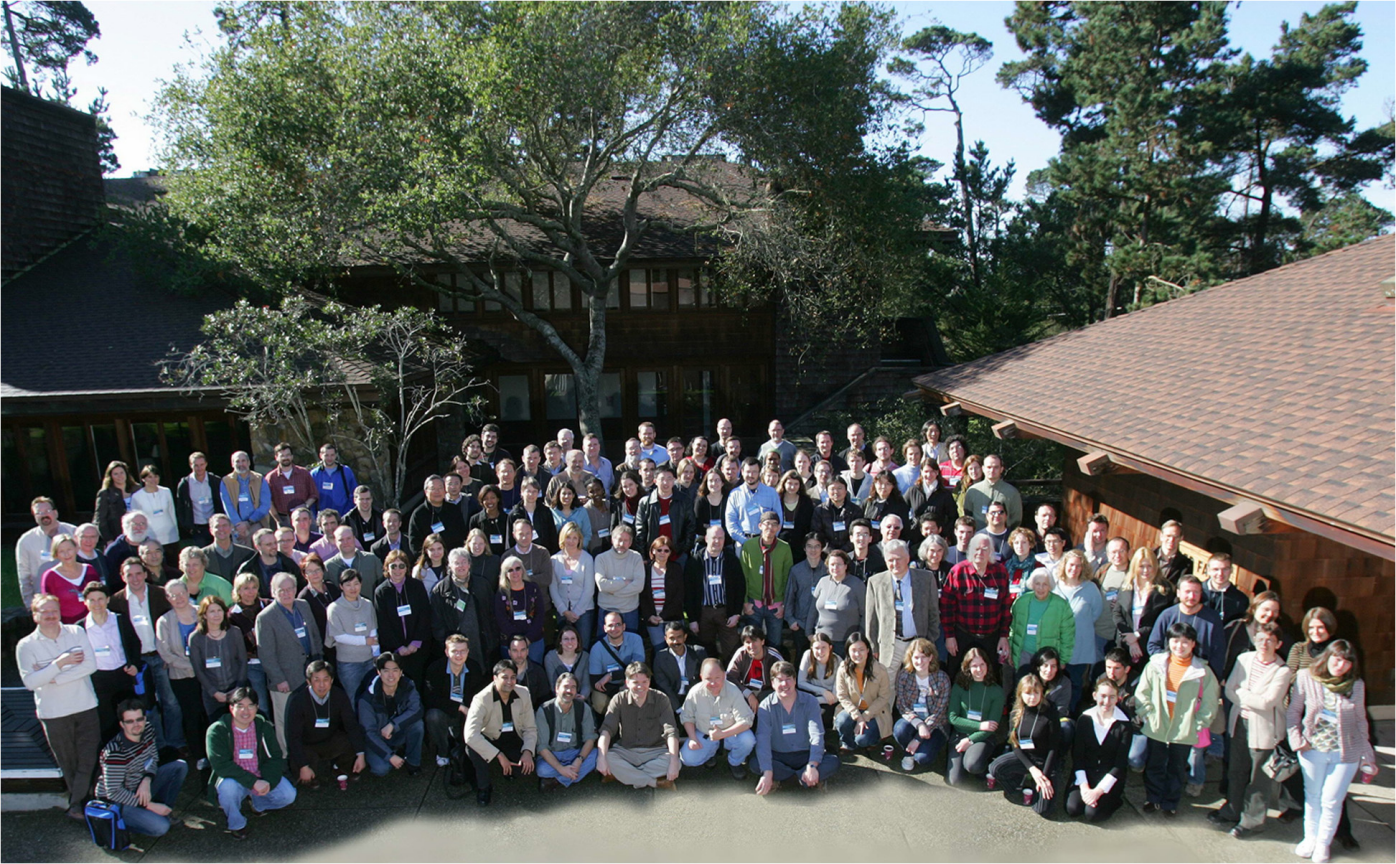
**A group photo from the 2nd International Conference/Workshop: “Genomic Impact of Eukaryotic Transposable Elements” held at the Asilomar Conference Center in Pacific Grove, CA, on 6 to 10 February 2009. **Roy Britten, in checkered shirt, next to Maxine Singer (on his left) and the organizer, Jerzy Jurka (on his right). The majority of the original members of the Editorial Board of Mobile DNA are also present in the photo, including two of the three co-founding editors, Nancy Craig and Thomas Eickbush.

The role of TEs in evolution remained Roy’s lifelong interest until his last paper in 2010 documenting more recent activity of Alu elements in the human genome when compared to other primate genomes [4]. A year earlier, he made a presentation entitled “History and Relationships of TEs” at the 2009 Asilomar conference on “Genomic Impact of Transposable Elements” (see the photo in Figure 1). His presentation focused on the role of TEs in the formation and control of eukaryotic genes. During the same session, the role of non-coding DNA in gene regulation was extensively discussed with ample reference to Roy’s pioneering work. He intended to participate in the 2012 conference, but sadly was diagnosed with pancreatic cancer in August 2011.

### Exaptations of transposable elements

In 1969, Roy Britten and Eric Davidson proposed an influential hypothesis for gene regulation in ‘higher cells’. The model merged concepts drawn from the seminal discovery that diverse eukaryotic genomes are replete with interspersed repetitive DNA [2] with the earlier, groundbreaking work of Jacob and Monod on the lactose operon of *Escherichia coli* and of McClintock on maize ‘controlling elements’. One of the pivotal ideas in the Britten-Davidson model was that interspersed repeats could distribute the same *cis*-regulatory elements to a ‘battery’ of genes scattered throughout the genome, allowing for the coordinated control of gene expression.

More than 40 years have elapsed since the formulation of the Britten-Davidson hypothesis and several talks at the meeting presented data in support of this prescient model. David Haussler (University of California Santa Cruz, CA, USA) presented data suggesting that the co-option of mobile elements for regulatory function is an ancient and pervasive phenomenon in the history of vertebrate genomes that has contributed innovations at various levels of cell signaling at different times during vertebrate evolution. Nori Okada (Tokyo Institute of Technology, Japan) discussed how a series of ancient exaptation events of multiple members of the AmnSINE1 family have contributed to the emergence of novel enhancers that control the expression of genes implicated in the development of brain, sensory, and craniofacial features unique to mammals. Okada argued that these exaptations were key to mammalian survival in a well-documented mass extinction at the Permian-Triassic boundary. Intriguingly, recent studies by Gill Bejerano (Stanford University, Palo Alto, CA, USA) also pointed to a dramatic enrichment of exapted AmnSINE1 family members near genes involved in mammalian corticogenesis. Petra Schwalie (EMBL-EBI, Wellcome Trust, Cambridge, UK) also presented data consistent with the Britten-Davidson model. She showed that several waves of SINE amplification at different times during mammalian evolution have dispersed thousands of binding sites for the insulator protein CTCF, a major organizer of genome architecture and regulation. Likewise, King Jordan (Georgia Tech University, Atlanta, GA, USA) presented evidence that MIRs, an ancient class of mammalian SINEs, commonly function as insulator elements in the human genome through a mechanism that likely is independent of CTCF binding. These exciting studies yield growing support to the visionary idea of Britten and Davidson that mobile elements have been a profuse source of new *cis*-regulatory elements, at least in mammalian genomes. The role of mobile elements in regulatory evolution is less clear in other organisms, but recent studies in Drosophila presented by Nikolai Tchurikov (Russian Academy of Sciences, Moscow, Russia) and by Josefa Gonzales (Institute of Evolutionary Biology, Barcelona, Spain) suggest that mobile elements have contributed adaptive regulatory mechanisms; however, these impacts may be harder to pinpoint because of the volatile nature of mobile element sequences in these genomes.

Another facet of mobile element exaptation is the ‘domestication’ of their gene product(s). Several classic and novel examples of this type of exaptation were discussed at the meeting. Jürgen Brosius (University of Münster, Germany) described the startling behavioral phenotypes of mice knockouts for BC1, a tRNA-derived retrogene, which is unusual because it has continued to give rise to new retroposition events long after its exaptation in a rodent ancestor. Lucas Gray (University of Washington, Seattle, USA) presented new functional data on the primate-specific PGBD3-CSB protein, which arose by fusion of a transposase to the Cockayne Syndrome B gene. He showed that the fusion protein has transcriptional activator properties and is tethered to hundreds of binding sites in the human genome derived from a related transposon family.

Not only retroposition-derived elements (mostly parts thereof) but also DNA transposons propagating in the “cut and paste” mode can contribute to exaptations. One of the most notable examples is the transposase-derived RAG recombinase, which was instrumental in the evolution of the adaptive immune system in jawed vertebrates [5]. Nancy Craig (Johns Hopkins School of Medicine, Baltimore, MD, USA) reported mechanistic evidence pointing to a transposase of the Transib superfamily as the progenitor of the RAG1 recombinase. She also described an active TE in the red flour beetle, as well as her ability to functionally resurrect a functionally related mammalian transposon.

As documented above, TEs also can contribute to the proteome of a cell. Casimir Bamberger (The Scripps Research Institute, La Jolla, CA, USA) used mass spectrometry to examine which of the many TEs contribute to the proteome. For example, he demonstrated that loss of the Piwi-like protein nvPiwil1 and UV irradiation in the starlet sea anemone induces translation of open reading frames with sequence similarity to TE sequences. Certain forms of stress also recruited TE proteins to the chromatin-associated proteome in human cells. Clearly, how TEs contribute to the proteome is an area for further exploration in the future.

### Other impacts of TEs

Despite these interesting cases of exaptations, we need to be mindful that TEs are first and foremost a tremendous mutational force. Several talks at the meeting illustrated the dazzling array of effects that TE insertions can have on the structure and expression of the genome and the deleterious consequences TE insertions can have on host genomes.

David Symer (Ohio State University Comprehensive Cancer Center, Columbus, OH, USA) used targeted deep sequencing to map endogenous retrovirus (ERV) insertions in the genomes of highly divergent mouse strains. Polymorphic insertions of young ERVs are relatively depleted from gene introns and especially from genes that are highly expressed in stem cells or are involved in development. These findings suggest that the ERVs are likely to have a deleterious impact on gene expression. Indeed, Symer described several cases (and perhaps at up to 100 or more genes across the genome) where intronic ERVs strongly trigger premature transcriptional termination at distances up to >12.5 kb upstream of the ERV elements. In heterozygous animals, the parent of origin of the ERV influences expression levels of the non-terminated gene transcripts, suggesting that the impact of ERVs upon certain transcripts can be regulated by epigenetic controls.

The subsequent talk by Dixie Mager (British Columbia Cancer Agency, Vancouver, Canada) examined the distribution of ERVs and other TEs within human and mouse introns. These analyses allowed predictions of what types of intronic TE insertions are most likely to be deleterious. Mager showed the following factors influence the mutational spectrum of TE insertions: a bias against proximity to splice sites, full-length versus truncated TEs, and TE orientation. Genes with extremely low or high TE densities were also highlighted in the presentation, and evidence was presented that actively transcribed genes in embryonic stem cells are more likely to be rich in TEs. These data suggest that the signature of initial insertion site preference may still exist for mammalian TEs.

Another major contribution discussed how mobile elements generate structural variation post-insertionally, through homologous recombination. Prescott Deininger (Tulane University Cancer Center, New Orleans, LA, USA) presented analyses from his laboratory based upon cell culture assays demonstrating the potential that mobile elements have to serve as scaffolds for homologous recombination events. Deininger emphasized the roles that sequence identity and cellular factors play in modulating the rate of recombination. For example, longer regions of sequence identity tend to promote more efficient recombination events between Alu elements. The combination of insertional mutagenesis by mobile elements, as well as the potential for post-insertion recombination, represents a powerful force acting to alter the genomic landscape of eukaryotic genomes.

Ben Koop (University of Victoria, Canada) presented analyses of the repeat element content of Salmonids. Salmonids represent rapidly evolving species complexes that are thought to be pseudotetraploid, though they may be in the process of reverting to a stable diploid state. The pseudotetraploid state presents a number of opportunities for evaluating the contribution of various repeat families to the overall structure and instability of the genome, as well as for the determination of the contribution of repeat sequences to speciation within the Salmonid species complex. The pseudotetraploid genome state, along with relatively high levels of repeated sequences, presents significant challenges for the accurate sequence assembly of the salmon genome sequence.

### Host/TE interactions

Due to the potentially harmful effects of TE acquisition and expansion, hosts have evolved mechanisms such as DNA methylation of TEs to control their “virulence” in the genome. Apparently, modes other than DNA methylation, such as covalent histone modification, play a key role in TE silencing. Matthew Lorincz (University of British Columbia, Vancouver, Canada) reported that several subclasses of ERVs are maintained in a silent state in mouse embryonic stem cells by histone H3 lysine 9 methyltransferase Setdb1/Eset. Interestingly, this transcriptional silencing is independent of DNA methylation status. In addition, Lorincz discussed RNAseq-based transcriptome analyses in mouse embryonic stem cells to identify the novel “chimaeric” genic transcripts that initiate in long terminal repeat (LTR) promoters and splice to genic exons.

To document the interplay between host defense mechanisms and the activity of retroposons, Michael Wilson (Cambridge Research Institute, UK) studied a model in which human chromosome 21 had been introduced into the mouse. This chromosome contains TEs that arose and spread in primates after they diverged from a common ancestor with rodents. Many repetitive elements on human chromosome 21 were hypomethylated at the DNA level and were aberrantly associated with histones harboring activating modifications in somatic and germline tissues. Hence, many dormant promoters, transcription factor binding sites, and insulators that are silenced in the human genomic background, are activated in the new genomic environment. It will be interesting to see, in long-term studies, whether some of the elements (in the case that master copies reside on human chromosome 21) spread to other mouse chromosomes and whether these elements ultimately become silenced in the mouse genome.

Erez Levanon (Bar-Ilan University, Israel) demonstrated that certain retrotransposons are likely to be frequent targets of editing by the APOBEC3 family of cytidine deaminases. The APOBEC3 protein family, by introducing C-to-U mutations in (-) strand cDNA (essentially fixing G to A mutations in the (+) strand of the retrotransposed cDNA), is thought to inactivate viruses and virus-like sequences by hyper-mutation. Thus, he posited that APOBEC3-dependent “DNA editing” may provide a potential mechanism for retrotransposon domestication.

Several reports focused on the role of small RNAs as central players in TE silencing. Andrea Schorn (Cold Spring Harbor Laboratory, NY, USA) investigated the role of small RNA-induced TE repression in embryonic and extra-embryonic tissues of mice. In embryonic and trophectoderm stem cells, 17-31nt RNA profiling revealed an increase in small RNA production for relatively young TE families, such as IAP, ERV-K, and ERV-L endogenous retroviruses. Small RNA levels were correlated with an abundance of bidirectional transcripts, but not with TE methylation status. Such small RNA-mediated post-transcriptional silencing may be driven by the necessity to suppress expression of active TEs in embryos, and to avoid embryo re-infection with retrovirus-like elements from the surrounding somatic tissues. Justin Blumenstiel (University of Kansas, Lawrence, KS, USA) presented an overview of the contribution of maternally transmitted piRNA to hybrid dysgenesis in *Drosophila virilis*. Contrary to earlier findings, he concluded that all chromosomes could possibly contribute to maternal protection against TE-mediated hybrid sterility. He also highlighted certain aspects of evolution of piRNA machinery in members of the genus Drosophila with varying TE content, reporting that these fast-evolving genes can develop codon biases that favor the increase in translational efficiency with the increasing transposon load, thereby avoiding titration of the silencing machinery by increasing piRNA quantities. Erin Kelleher (Cornell University, Ithaca, NY, USA) compared piRNA abundance in *D. melanogaster, D. simulans,* and their respective inter-specific hybrids. While the difference between piRNA pools in these species was evident, there was no correlation between maternal deposition and TE silencing in hybrid offspring. Rather, she observed global de-repression of TEs originating from either parent, presumably due to the failure of species-specific piRNA machinery to adapt to novel environments. Fernando Rodriguez (MBL, Woods Hole, MA, USA) reported an increase in relative abundance of pi-like RNAs in bdelloid rotifers subjected to a dose of ionizing radiation that would cause hundreds of DNA double strand breaks per genome. These small RNAs predominantly mapped to low copy number TEs, were mostly recovered in anti-sense orientation, and exhibited strong 5’-uridine bias.

In plants, Damon Lisch (University of California, Berkeley, CA, USA) demonstrated that silencing of the autonomous maize *MuDR* DNA transposon can be mediated by small RNAs. When SGS3, a key component of a silencing pathway required for the production of trans-acting RNAs (tasiRNAs), is downregulated, it leads to loss of transcriptional silencing due to absence of DNA methylation. In maize, SGS3 downregulation occurs in transition leaves just as the plant is preparing to enter reproductive growth, suggesting a connection between vegetative phase transition in plants and epigenetic regulation of TEs. Lisch has also demonstrated that maize genes can be co-opted into the tasiRNA-silencing pathway following insertion of TEs, resulting in a high level of plasticity with respect to gene regulation.

### Transposable elements in asexually propagating species

It has long been thought that the reproductive mode of an organism can influence its TE content, which has stimulated comparisons between sexually and asexually reproducing species. Irina Arkhipova (MBL, Woods Hole, MA, USA) described a multilayered system of genome defense against TEs in asexual bdelloid rotifers. She demonstrated that these defenses include, but are not limited to, small RNA-induced TE silencing, deletion via homologous recombination and microhomology-mediated end-joining, and insertion of asparagine-rich segments within coding sequences of certain TEs. At the same time, bdelloid genomes harbor at least two types of single-copy reverse transcriptase-related genes, one that belongs to *Penelope*-like elements (PLEs) and may play a role in telomere maintenance, and another that belongs to a previously unknown class named *rvt* genes, found in a wide but patchy collection of organisms. Eugene Gladyshev (Harvard University, Cambridge, MA, USA) described his initial biochemical and genetic characterization of the RVT protein of *Neurospora crassa*, an active representative of this mysterious group of reverse transcriptase-related cellular genes. The *N. crassa* RVT protein is non-essential for growth, but is dramatically upregulated when protein synthesis is inhibited *in vivo*. The RVT protein also exhibits robust terminal transferase activity *in vitro*. Ken Kraaijeveld (Leiden University, The Netherlands) further explored the theme of asexuality and its influence on TE content. He used next-generation Illumina sequencing to compare TE abundance in genetically distinct sexual and asexual lineages of the parasitoid wasp *Leptopilina clavipes*. Asexuality in this wasp was recently induced by Wolbachia infection, which may have led to an increase in copy numbers for several DNA TEs and one gypsy-like LTR TE. However, despite the presence of the *Dnmt1* gene in *L. clavipes*, gypsy methylation was not observed in either lineage. Jens Bast (Georg-August-University, Göttingen, Germany) used a similar approach for comparisons between four taxa of oribatid mites, two of which are sexual and two are long-term parthenogens. In this case, long-term asexuals appeared to display reduced TE loads in comparison with sexuals. Finally, Sarah Schaack (Reed College, Portland, OR, USA) argued that there are several opposing forces that may cause either decreases or increases in TE copy numbers in sexually and asexually reproducing populations of the cyclically parthenogenetic crustacean, *Daphnia pulex*. She further stressed that it may often be difficult to discriminate between forces exerting the most influence. On balance, recently developed asexuality may not allow sufficient time to affect TE content significantly, while long-term asexuality may be expected to leave its imprint on TE diversity and abundance.

### Transposable elements and population genetics

The population genetics session was opened by Dmitri Petrov (Stanford University, Palo Alto, CA, USA) who presented an overview of Drosophila TE dynamics in natural populations and the major forces governing the distribution and behavior of 1824 diverse TEs in genomic sequences of 158 individual or pooled Drosophila strains. In agreement with earlier findings, most TEs were found at low population frequencies and were largely subject to purifying selection against deleterious products of ectopic recombination, rather than against deleterious effects on neighboring genes or against over-expression of TE-encoded products. Cristina Vieira (CNRS, Lyon, France) addressed the problem of TE control in natural populations by studying germline piRNA-mediated silencing of endogenous Tirant retroviruses in *D. melanogaster* and *D. simulans*, which are present in the latter species in much smaller copy numbers. She argued that the levels of variation in piRNA machinery could contribute substantially to the observed variation in TE copy number. The accelerating speed of accumulation of next-generation sequencing data will undoubtedly dominate population genetic studies in the near future.

### Inter- and intra-individual transposable element variation

One of the major themes of the meeting was in the characterization of the various ways that transposons impact the genome. Individual transposons serve as insertional mutagens in both the germline and somatic tissues, but they can also serve as additional sources to generate structural genetic variation through post-insertion recombination based processes. Kyle Upton (University of Edinburgh, UK) presented a new approach to identify newly integrated somatic retrotransposable element insertions called “retrotransposon capture sequencing”. Using this approach, thousands of somatic L1 and Alu element insertions have been identified in neuronal tissues. These data suggest the overall importance of transposons in creating somatic structural variation. Scott Devine (University of Maryland, Baltimore, MD, USA) discussed the amount of TE-related variation that can be obtained using high throughput second generation sequencing approaches. He reported on the movement of transposons in certain lung tumors, suggestive once again of the contribution that these elements make to somatic mutagenesis. In addition, Devine discussed ongoing efforts to make risk estimates for L1-based mutagenesis on the basis of the number of “hot” L1 drivers, expanding upon some of the initial pioneering research on allelic variation in retrotransposition performed in the Kazazian and Moran laboratories. Adam Ewing (Center for Biomolecular Science and Engineering, UCSC, CA, USA) reported the use of sequence comparisons involving normal human tissue with a large number of different tumors to detect hundreds of novel TE integrations in somatic cells. To this end, existing algorithms were supplemented with novel computational approaches and applied to The Cancer Genome Atlas whole-genome datasets. Hopefully, this information will not only yield insights with respect to TE expansion in somatic tissue, but also might be relevant for understanding insertional changes leading to, and accompanying, the clonal evolution of tumor cell populations. Mark Batzer (Louisiana State University, LA, Baton Rouge, USA) also discussed the overall contribution of mobile elements to genomic diversity in the human 1000 genomes project. Thousands of new mobile element insertion polymorphisms have been identified in the pilot phase of these studies. Sue Wessler (UC Riverside, CA, USA) presented the results from second-generation analyses of rice genomes. She reported on the burst of MITE movement that occurred and the levels of associated insertion polymorphism.

All the above studies present new high throughput approaches for the detection of newly inserted transposons. As more of the studies are completed, the mode and tempo of transposon proliferation in different tissues within an individual and in different eukaryotes will be elucidated. These studies truly represent insights into TE mobilization on a previously unprecedented scale.

### Computational identification of transposable elements and discoveries

A series of talks presented the next frontier for the computational identification of TEs. At the core are still pipelines or networks that reliably recognize and characterize transposed elements provided by well-founded tools such as RepeatMasker and RepBase. However, it is important to note that these tools are not static entities, but rather are adaptive components in need of regular improvement to complement the emerging requirements of growing genome information. Arian Smit (Institute for Systems Biology, Seattle, WA, USA) and Travis Wheeler (HHMI Janelia Farm Research Campus, Ashburn, VA, USA) presented a novel approach to detect highly diverged ancient TEs using hidden Markov models (HMMs) implemented in the software package HMMER3. This probabilistic inference technology was originally introduced for protein searches. The reconstruction of ancestral genomes and the consideration of genomes with low substitution rates, such as the crocodile genome (also reported by Andrew Shedlock), are ideal additional strategies for digging even deeper into the past of retroposed elements. As a predictor, HMMER surpasses the performance of the RepeatMasker-implemented, single-sequence homology search tools cross_match and blastn. Smit demonstrated the potential utility of this approach by detecting more and more complete highly diverged test “ghost” repeats. David Pollock (University of Colorado, Denver, CO, USA) then briefly discussed an alternative approach for the identification of all repeated DNA sequences using “P-clouds”, and presented a refined alternative to the “master gene” model to explain the expansion of some families of transposons. A prediction with the aid of P-clouds suggested that more than two thirds of the human genome is derived from TEs! Overall, these talks provide exciting new computational approaches for detection and analysis of repeated DNA sequences.

All known “cut-and-paste” DNA transposons encode only one to two conserved proteins. On the other hand, “self-synthesizing” Polintons encode up to eight conserved proteins. It is believed that such complexity is due to evolution of Polintons from DNA viruses. Vladimir Kapitonov (Genetic Information Research Institute, Mountain View, CA, USA), reported two groups of fungal DNA transposons, Entons and Intons, that encode five to six conserved proteins and evolved from their En/Spm and IS3EU “cut-and-paste” ancestors >100 million years ago. Therefore, some complex DNA viruses might have evolved from simple DNA transposons through explosion of their complexity. He also presented diverse DNA transposons from different super-families that harbor conserved proteins acquired from a wide range of host proteins, suggesting that the latter are involved in regulation of transposition.

### Transposable elements as phylogenetic markers

Retrotransposed elements accumulate over millions or hundreds of millions of years and encrypt valuable information about the evolution of their organism. Some eventually might influence the fitness of the species harboring them, but most are neutral hitchhikers throughout the persisting stream of life.

Mark Batzer (Louisiana State University, Baton Rouge, LA, USA) reported on several thousand such young, polymorphic hitchhikers his group discovered at the species level within the framework of the human 1000 Genomes Project and compared these insertion polymorphisms to other mobile element insertion polymorphisms previously uncovered from PCR display studies. From steps deeper along the tree of primates, he used mobile element insertions as clade markers to understand the early evolution of gibbons, surprisingly placing the Siamang (*Symphalangus syndactylus*) at the base of the gibbon phylogeny.

Lucia Carbone (Oregon Health and Science University and Oregon National Primate Research Center, Portland, OR, USA) and Annette Damert (Babes-Bolyai University, Cluj-Napoca, Romania) discussed their studies of a newly identified family of composite non-autonomous non-LTR retrotransposons in the gibbon genome termed LAVA elements. Carbone demonstrated that LAVA elements are involved in centromere related remodeling in Hoolock gibbons. The expansion of LAVA elements within the gibbon genome is a hallmark of the increased genomic plasticity in the gibbon lineage and contributes to the enhanced structural variation within the gibbon lineage. Damert discussed her studies of LAVA, PVA, and FVA elements. These elements share structural features with SVA elements (see Gerald Schumann below). Interestingly, PVA and FVA have acquired additional sequences at their 3’ ends by RNA splicing (derived from exon 4 and part of intron 4 of PTGR2 and from a free right Alu monomer (FRAM)). It will be interesting to develop a cell-based assay to assess whether LAVA elements retrotranspose in cultured cells and how the variant 3’ ends of PVA and FVA elements influence retrotransposition.

Jürgen Schmitz (University of Münster, Germany) presented retroposon-based reconstructions of 160 million-year-old phylogenies that are close to the boundary of resolution. He illustrated a range of phylogenetically informative retrotransposon presence/absence patterns in mammals and birds as well as the advantage of such a marker system to distinguish sequence divergences and ancient polymorphisms. Retrotransposons not only encrypt information about species phylogeny, but also indirectly convey information about their biogeography, sex determination, or brain evolution. He discussed how using transposed elements as phylogenetic markers and for other reconstructions requires very careful comparative evaluations of presence/absence loci, multidirectional screening strategies, and stringent statistical evaluations of the results.

Andrew Shedlock (College of Charleston, SC, USA) provided the first glimpse of new genomic information and molecular evolution of TEs in the Anolis lizards and painted turtle genomes compared to other large-scale reptilian genome sequences and their mammalian sister groups. He demonstrated that having such comparative reptile genome information is essential to understand the retrotransposon landscape and distribution signals in amniotes and in vertebrates generally.

In the age of genomics, reliable automated tools to explore the depths of species evolution are indispensable. Wojciech Makalowski (University of Münster, Germany) presented new automated approaches, including the transposition in transposition (TinT) strategy to reliably derive the historical succession and activity patterns of retrotransposon families and the TEclass program to classify such elements. He also discussed different approaches for the detection and biological analyses of TEs with emphasis on pipelines that enable a streamlined evaluation of the historical content of retrotransposable elements.

With the exception of monotremes, the retrotransposon landscape of mammals is significantly shaped by LINE-1 and associated co-mobilized elements. Holly Wichman (University of Idaho, Moscow, ID, USA) illustrated the importance of LINE-1 retroposition and described its potential functional role in genome integrity. Interestingly, a screen of over 200 mammalian species from almost all eutherian and marsupial clades revealed the extinction of LINE-1 elements in only a few cases. Such examples are exciting scenarios for understanding a future without active LINE-1 elements.

### Transposable element mechanisms

Several talks discussed mechanistic aspects of TE mobility. John Moran (University of Michigan, Ann Arbor, MI, USA) built on previously published findings, which demonstrated that endonuclease-deficient LINE-1 elements could use dysfunctional telomeres as integration substrates in Chinese hamster ovary cells lacking a protein important for the non-homologous end-joining pathway of DNA repair (DNA-protein kinase catalytic subunit (DNA-PKcs)). He demonstrated that LINE-1 ribonucleoprotein (RNP) preparations could use oligonucleotides that mimic the G-rich overhang present at telomeric ends as substrates to initiate LINE-1 mRNA reverse transcription. These RNP preparations also contain a nuclease activity that could process oligonucleotide adapters before being used to prime LINE-1 mRNA reverse transcription. Whether the nuclease activity is attributed to the LINE-1 encoded proteins or an associated cellular protein remains an open question. However, these findings further highlight mechanistic similarities between an alternative endonuclease-independent pathway of LINE-1 retrotransposition and telomerase action.

Haig Kazazian (Johns Hopkins Medical School, Baltimore, MD, USA) discussed recent efforts, which use photoactivatable-ribonucleoside-enhanced cross-linking and immunoprecipitation (PAR-CLIP), to identify cellular RNAs that interact with LINE-1 ORF1p in HEK293T cells. He found that ORF1p binds unstructured regions in non-autonomous retrotransposons (SVA and Alu RNA), as well as the 3’-UTR and last exons of a wide variety of cellular mRNAs. It will be interesting to examine whether low concentrations of ORF1p binding are required for Alu and/or SVA retrotransposition, and if ORF1p binding to cellular mRNAs is correlated with processed pseudogene formation.

Gerald Schumann (Paul Ehrlich Institut, Langen, Germany) showed that the human LINE-1 encoded proteins (ORF1p and ORF2p) could act in *trans* to promote the faithful retrotransposition of genetically tagged (SINE-VNTR-Alu) SVA elements in cultured human HeLa cells. Similar work also was reported from the Kazazian lab. Interestingly, members of the human-specific SVAF1 subfamily, which acquired a 5’ MAST2 sequence by RNA splicing and a 3’ AluSp sequence by 3’ transduction, are *trans*-mobilized by the L1 protein machinery approximately 25-fold more efficiently than the parental element lacking the AluSp sequence. Clearly, this retrotransposition assay should allow the rapid identification of *cis*-acting sequences within SVA elements that are important for its transcription and retrotransposition.

Anthony Furano (National Institutes of Health, Bethesda, MD, USA) discussed recent biochemical experiments using recombinant human LINE-1 ORF1p. First, he showed that, in the absence of nucleic acids, ORF1p polymerizes in the very low salt conditions that are optimal for nucleic acid binding. The ORF1p C-terminus mediates polymerization, and the addition of nucleic acid rapidly allows the ORF1p polymer to resolve into homotrimers. Second, he demonstrated that ORF1p has a biphasic nucleic acid chaperone activity. ORF1p initially recognizes mismatched strands in a duplex as single stranded DNA and protects the duplex from melting. However, when present in the polymerized form, ORF1p subsequently promotes melting of the same DNA substrate. These data have important implications in understanding how ORF1p interacts with nucleic acids. Moreover, the ability of ORF1p to polymerize may prevent it from diffusing into the cytoplasm, providing a plausible mechanism for the *cis*-preferential binding of ORF1p to LINE-1 mRNA.

Finally, Zuzsanna Izsvak (Max-Delbrück-Center for Molecular Medicine, Berlin, Germany) reported that the heat shock (HS) response machinery of the host is regulating transposition of the DNA transposon Sleeping Beauty (SB). SB harbors conserved transcription factor binding sites for HS factor 1 and 2 (Hsf1/2), which induce transcription of the transposase gene. SB is a vehicle for distribution of HS factor binding sites in the host genome. Furthermore, the transposase protein product, albeit stable, is sensitive to mis-folding; hence, the HS response might activate latent SB integrations and thus trigger transposition.

### Role of transposable elements and viruses in horizontal gene transfer

In early evolution, the Darwinian Threshold (the transition period between the early stages of evolution when horizontal gene transfer was universal and the era when separate species began to exist) was not in place yet, with rampant exchange of genetic material [6]. Even today, these barriers are much more penetrable than originally thought. The power of comparative genomics reveals frequent exchange of genes (or parts thereof) between cellular life forms, TEs, and viruses. As Eugene Koonin (NCBI, NLM, NIH, Bethesda, MD, USA) has presented, this is particularly apparent in giant viruses, such as mimiviruses that harbor a genome in excess of one megabase. These and other viruses harbor their own viral parasites as well as their own mobile elements that may facilitate horizontal gene transfer.

A similar scenario was also borne out in the talk of Cedric Feschotte (University of Utah, Salt Lake City, UT, USA), who stressed that horizontal gene transfer not only plays a role in bacterial evolution, but also plays a role in eukaryotic evolution. Two vehicles of horizontal transfer are significant. Transposons are not only moving between loci in individual genomes and then are propagated vertically, but also are crossing species barriers, spawning huge waves of lineage-specific invasions in “naive” hosts. Another route of horizontal transfer is the viral one. Not only the well-known endogenous retroviruses, but almost every major type of virus can be integrated into host genomes. Thus, when jumping species barriers, viral genes offer potential fodder for exaptation.

### Concluding remarks

The series of the Asilomar meetings “Genomic Impact of Eukaryotic Transposable Elements” has earned a distinctive place among other meetings in the field of mobile DNA, such as the FASEB Summer Research Conference “Mobile elements in mammalian genomes”, the ASM Conference on Mobile DNA, and the International Congress on Transposable Elements in France. It attracts researchers from all over the world and brings together computational biologists working on genome annotations and experimental scientists investigating various aspects of transposon biology. Such interactions serve as a catalyst for new ideas and lead to new discoveries both by theoreticians analyzing vast amounts of experimental data and by experimentalists seeking to uncover and validate novel biological phenomena emerging from *in silico* findings. We thank the organizer, Jerzy Jurka, for bringing this community of scientists together to interact and discuss their findings. The meeting was incredibly successful and we eagerly await the next meeting of this highly interactive group of scientists.

## Abbreviations

ERV: endogenous retrovirus; HMM: hidden Markov model; HS: heat shock; LTR: long terminal repeat; RNP: ribonucleoprotein; SB: Sleeping Beauty; tasiRNA: trans-acting RNA; TE: transposable element.

## Competing interests

The authors declare that they have no competing interests.

## Authors’ contributions

IA, MB, JB, CF, JVM, JS, and JJ all wrote an approximately equal number of meeting presentation summaries. All authors read and approved the final manuscript.
